# Abnormal cerebellar activity and connectivity alterations of the cerebellar-limbic system in post-stroke cognitive impairment: a study based on resting state functional magnetic resonance imaging

**DOI:** 10.3389/fnins.2025.1543760

**Published:** 2025-03-19

**Authors:** Haiyi Zhang, Juan Lu, Lu Zhang, Jidan Hu, Jiajun Yue, Yunhan Ma, Qi Yao, Pingping Jie, Min Fan, Jiliang Fang, Jie Zhao

**Affiliations:** ^1^Department of Magnetic Resonance Imaging, The Affiliated Traditional Chinese Medicine Hospital, Southwest Medical University, Luzhou, Sichuan, China; ^2^Department of Acupuncture and Rehabilitation, The Affiliated Traditional Chinese Medicine Hospital, Southwest Medical University, Luzhou, Sichuan, China; ^3^Department of Radiology, The Second People’s Hospital of Neijiang, Southwest Medical University, Neijiang, Sichuan, China; ^4^Department of Nuclear Medicine, The Affiliated Hospital of Southwest Medical University, Luzhou, China; ^5^Molecular Imaging Key Laboratory of Sichuan Province, Department of Nuclear Medicine, The Affiliated Hospital of Southwest Medical University, Luzhou, China; ^6^Department of Radiology, The Affiliated Traditional Chinese Medicine Hospital, Southwest Medical University, Luzhou, China; ^7^Guang’anmen Hospital, China Academy of Chinese Medical Sciences, Beijing, China

**Keywords:** stroke, mild cognitive impairment, cerebellum, functional magnetic resonance imaging, limbic system

## Abstract

**Background:**

Stroke is an important cause of cognitive impairment. Post-stroke cognitive impairment (PSCI) is a prevalent psychiatric disorder following stroke. However, the effects of PSCI on the cerebellum remain mostly unknown.

**Methods:**

A total of 31 PSCI patients and 31 patients without cognitive impairment after stroke were included in this study. The Mini-Mental State Examination (MMSE) and the Montreal Cognitive Assessment (MoCA) were administered to all participants. Analyses of ALFF, fALFF, and ReHo were employed to investigate alterations in brain neuronal activity, while limbic connectivity analysis was utilized to reflect changes within the abnormal connections within brain regions.

**Results:**

We found that ALFF values were increased in Cerebelum_7b_R, Cerebelum_Crus1_L. fALFF values were increased in Vermis_3. The ReHo values were increased in Cerebelum_8_R, Cerebelum_Crus2_R, Cerebelum_Crus1_L. The functional connection between Frontal_Mid_Orb_L and Cerebelum_Crus2_R brain regions was decreased. The functional connection between Hippocampus_L and Cerebelum_Crus2_R brain regions was decreased. The functional connection between Vermis_3 and Frontal_Med_Orb_L brain regions was decreased.

**Conclusion:**

The severity of cognitive impairment may influence the extent of functional connectivity disruption between the cerebellum and the limbic system. Furthermore, atypical alterations in neuronal activity within cerebellar regions are associated with cognitive decline.

## 1 Introduction

Stroke, a disorder that affects the cerebrovascular system and can result in significant disruptions in blood flow to the brain, can be caused by a number of reasons, which is the second most prevalent cause of death worldwide and the leading cause of adult disability (Chen et al., 2024). It has brought an extremely serious burden to society. Over 12.2 million new cases of stroke occur each year (Feigin et al., 2024). Globally, 25% of individuals above the age of 25 will experience a stroke throughout their lifetime (Feigin et al., 2024).

Cerebrovascular disease resulting from stroke may substantially affect patients’ ability for self-care in daily life, their executive function, and overall cognitive capabilities following recovery. Research indicates that individuals who have experienced a stroke face a 5–8 fold increased risk of developing cognitive impairment compared to the general population (Krishnamurthi et al., 2020). The development of post-stroke cognitive impairment (PSCI) is often correlated with the characteristics of the cerebrovascular lesions involved (Onyike, 2006). Stroke is recognized as a major contributor to PSCI (O’Brien, 2015). However, cognitive impairment often co-occurs with numerous complications during the early stages of stroke where symptoms tend to be relatively subtle, and because many patients possess limited understanding of their condition, effective treatment remains elusive for most individuals. Currently, there is a significant increase in the number of patients experiencing cognitive impairment. Most diagnoses rely heavily on clinical scales for evaluation (Regier et al., 2012). The concomitant and insidious progression of this disease alongside limitations inherent in biomarkers greatly complicates clinical diagnosis and treatment efforts.

Research has demonstrated that neuronal activity and network connectivity within the cerebellar regions of individuals who have experienced a stroke are significantly disrupted, with this disruption being closely linked to the severity of the condition (Pantoni, 2010). Thomasson et al. (2019) found that irregularities in the right cerebellar lobes VIIb, VIIIa, b, and IX were associated with emotional alterations, indicating that the cerebellum may also contribute to cognitive functions. A recent longitudinal neuroimaging study revealed that cerebellar hypoplasia resulting from cerebellar hemorrhage in preterm infants serves as an independent structural biomarker predictive of cognitive dysfunction in pediatric populations, underscoring the cerebellum’s critical role in cognitive development (Drommelschmidt et al., 2025). Significant alterations in cerebellar activation were observed during both positive and negative emotional states, suggesting that cerebellar activity exerts a substantial influence on higher-order cognitive functions, including emotion regulation (Oboshi et al., 2025). This further implies that changes in cerebellar activity can impact overall cognitive performance. Chen et al. (2025) similarly discovered that alterations in the cerebellar-brain connectivity can significantly influence cognitive function and emotional state. Additionally, several studies emphasized that the cerebellum served as a crucial component within the distributed cortico-subcortical neural circuitry involved in cognitive manipulation (Schmahmann, 1997). Collectively, these findings underscored the cerebellum’s substantial involvement in human cognitive functions and suggested potential neurobiological mechanisms that might underlie PSCI.

Resting-state functional magnetic resonance imaging (rs-fMRI) has proven to be a valuable tool in the investigation of the neurobiological underpinnings of various diseases (Zhang et al., 2024). Current literature indicates that rs-fMRI, by capturing spontaneous neural activity patterns in the brain during resting states, offers a distinctive perspective for elucidating the neuronal activity abnormalities and functional disruptions that contribute to cognitive impairments associated with stroke. Some recent investigations have demonstrated that neuroimaging studies can validate that cerebellar activity in stroke patients is associated with the onset of cognitive impairment. Duttagupta et al. (2024) employed the resting state brain network to show the poor overall efficiency of the default mode network (DMN) and dorsal attention network (DAN) in patients with cerebellar stroke. Employing ALFF and fALFF values, Zeng et al. (2024) discovered that stimulating the cerebellum enhanced balance and alleviated movement disorders in stroke patients. However prior investigations have not thoroughly studied the alterations in cerebellar neuronal activity among individuals experiencing both stroke and cognitive deficits. The present study aims to explore the changes in neural activity within the cerebellar regions of stroke patients who exhibit cognitive impairment, as well as to assess the connectivity between these cerebellar regions and the brain systems.

Therefore, we envision that PSCI is linked to a unique pattern of changes in the cerebellar neuronal system. We performed a comparison between two groups—one of stroke patients with minor cognitive impairment and the other of stroke patients without cognitive deficits—to evaluate this hypothesis. In order to provide important insights into the pathophysiology of early PSCI, our investigation aims to clarify its distinctive features. Furthermore, our goal is to clarify the patterns of changes in cerebellar neuronal activity linked to early cognitive deficits, finding potential imaging biomarkers that could predict the onset of cognitive impairment based on our findings, and look into the changes in connectivity between the cerebellum and other brain regions in the context of cognitive impairment.

## 2 Materials and methods

### 2.1 Participants and inclusion and exclusion criteria

This research encompassed data collected from September 2023 to September 2024, involving MRI assessments of 31 PSCI patients (S-MCI) and 31 stroke patients without cognitive impairment (S-C). *The inclusion criteria were as follows*: (1) a confirmed diagnosis of stroke by a neurology specialist within the preceding year, accompanied by the presence of bilateral paraventricular and basal ganglia lesions; (2) right-handedness; (3) the age range of 40–60 years; (4) scores on the Mini-Mental State Examination (MMSE) indicating illiteracy at 16–17 points, primary school education at 18–20 points and junior high school and above at 21–23 points; (5) scores on the Montreal Cognitive Assessment Scale (MoCA) consistent with a diagnosis of mild cognitive impairment; (6) no history of late-night activities, alcohol consumption, or intake of stimulating substances such as tea or coffee within 2 days prior to the MRI examination.

*Exclusion criteria include*: (1) contraindications to MRI; (2) history of cognitive impairment unrelated to stroke, other neurological deficits impacting brain function; (3) no cerebellar damage due to stroke. Participants with multiple strokes, characterized by bilateral lateral ventricle and basal ganglia lesions, were included in the study as well. PSCI were diagnosed by a neurologist and met the cognitive impairment diagnostic criteria outlined in the Diagnostic and Statistical Manual of Mental Disorders, Fifth Edition (DSM-V).

All study participants provided written and informed consent, and the study was approved by the Ethics Committee of the Affiliated Hospital of Traditional Chinese Medicine of Southwest Medical University (KY2021082-FS01).

### 2.2 Multiparameter fMRI scanning and data acquisition

In this study, the 16-channel head and neck joint coil of Siemens Skyra 3.0 T MRI was employed for data collection. T1W, T2W, and T2-FLAIR scans were conducted before the collection of resting state data, so as to exclude patients with cerebral hemorrhage and tumors and enable them to adapt to the scanning environment. The gradient recalled echo (GRE) sequence was employed to collect BOLD images, with the following parameters: repetition time (TR) = 2,720 ms, echo time (TE) = 40 ms, flip angle = 90°, thickness/gap = 4.0/0 mm, field of view (FOV) = 240 mm × 240 mm, in-plane resolution = 64 × 64, axial slices, acquisition time point 270, and acquisition duration of 12 min and 20 s. Additionally, anatomical T1-weighted whole brain magnetization-prepared rapid gradient-echo (MPRAGE) images could be configured with the following parameters: repetition time (TR) = 1960 ms, echo time (TE) = 2.98 ms, flip angle = 90°, thickness/gap = 1.0/0 mm, field of view (FOV) = 256 mm × 256 mm, in-plane resolution = 256 × 256, and the number of sagittal surface layers was 176.

### 2.3 Data preprocessing

Data preprocessing were conducted as previous study (Zhang et al., 2024). In briefly, we employed Restplus V1.28 software based on the MATLAB R2023b platform for processing. (1) The data of the first 10 time points were removed, and the fMRI data of the subsequent 190 time points were included in the analysis. As the scan commences, there will be fluctuations to adapt to the scanning environment, resulting in an unstable magnetic field. Therefore, the data of the first 10 time points should be removed to minimize or eliminate the influence of these data on the results. (2) Slice timing: Ensure the acquisition and acquisition time of all layers of the brain were consistent. (3) Realign: In this experiment, subjects with a head motion shift >2.5 mm and a rotation movement >2.5° were excluded. (4) Spatial normalization: Each subject’s image was registered and segmented with the T1-weighted whole-brain magnetization-prepared rapid gradient-echo (MPRAGE) images, and then the segmented gray matter was registered with the MNI standard template. (5). Subsequently, the functional image after spatial standardization was resampled to a voxel size of 3 mm × 3 mm × 3 mm.

### 2.4 Multiparameter fMRI data calculation

#### 2.4.1 ALFF and fALFF calculation

The values of ALFF and fALFF were calculated through the Restplus V1.28 software.^1^ Basedonthe data preprocessing, a spatial smoothing with an isotropic Gaussian kernel of 6 mm full width at half maximum (FWHM) was utilized for spatial smoothing to eliminate the linear drift caused by machine heating. Nuisance covariates regression was removed, including the average white matter signal, the average cerebrospinal fluid signal, and total head movement. By employing the fast Fourier transform, the time series of each voxel filtered in the subject’s brain was converted into a spectrum, and the power spectrum was obtained. The square root of the power spectrum within the range of 0.01–0.08 Hz was calculated and regarded as the ALFF. Then, the ALFF for each voxel was divided by the global average of the ALFF to obtain the normalized zALFF. The amplitude of the full band was calculated and normalized. The amplitude of ALFF was divided by the amplitude of the full band to obtain fALFF, and the same method was used to acquire the standardized zfALFF.

#### 2.4.2 ReHo calculation

Similarly, we carried out Detrend and 0.01–0.08 Hz filtering on the preprocessed images. The Kendall coefficient of Consistency (KCC) was calculated to measure the similarity of the time series of a given voxel to the time series of its closest 26 voxels. By computing the KCC of each voxel in the entire brain, a single homology was generated. To reduce the impact of individual KCC value differences on the KCC value, the KCC value of each voxel was divided by the average whole brain KCC value to normalize the ReHo plot. The KccReHo value of each voxel of all subjects was divided by the average ReHo value in the whole brain Mask to obtain a standardized KccReHo plot (zKccReHo). After Gaussian smoothing [with a smoothing kernel of (6 6 6)], the szKccReHo plot was obtained for subsequent statistical analysis.

#### 2.4.3 FC calculation

We extracted the brain regions that showed changes in ALFF, fALFF and ReHo analysis as areas of interest (ROI). After Detrend, Nuisance covariates regression and 0.01–0.08 Hz filtering, standardized zFC is obtained for subsequent statistical analysis.

### 2.5 Statistical analysis

We employed IBM SPSS 26.0 statistical software (IBM, Chicago, IL, United States) to analyze the fundamental data of the two groups of subjects. Age following a normal distribution was tested through the *T* (*t*) test of independent samples. Other measurement data (years of schooling, PSQI, ISI, and HAMD-24 scale scores) were evaluated by the rank sum test (Mann–Whitney *U* test). SPM12.0 software (Statistical Parametric Mapping 12)^2^ was utilized to conduct a two-sample *T-*test on the aforesaid calculated fMRI data, and the FWE method was chosen for multiple comparison correction. After correction, the region with *p* < 0.05 was defined as the region of statistical significance. The Montreal Neurological Institute (MNI) coordinates were adopted to locate the peak points in the regions with statistically significant differences. The range of brain regions where the peak points were situated was selected and named as the brain regions with statistically significant differences. GRETNA software^3^ was applied for NBS correction, and the functional connection of FC values was counted. Finally, Pearson correlation was utilized to analyze the correlation between these brain regions and the scores of PSQI, ISI, and HAMD-24.

## 3 Results

### 3.1 Clinical data analysis

**Figure 1:**
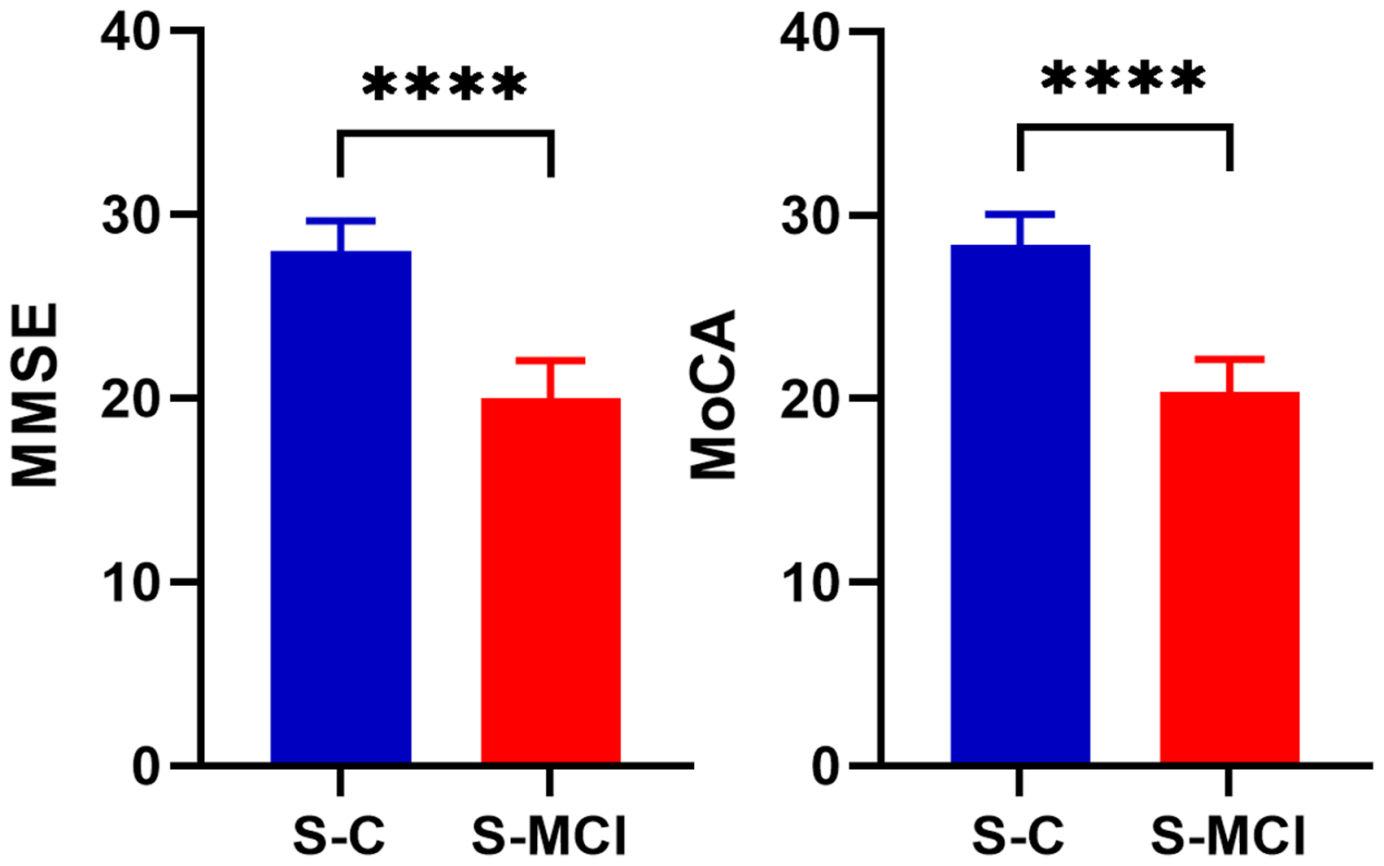
The bar chart shows that there are significant statistical differences in MMSE and MoCA values between S-MCI group and S-C group. ****p < 0.0001.

The study included 31 S-MCI and 31 S-C participants. The results showed that there was no significant difference in sex, age and education level between the S-MCI group and the S-C group (*p* > 0.05). The MMSE scale and MoCA scale scores of the S-MCI group were significantly lower than those of the control group, with statistical significance (*p* < 0.001) (Table 1 and Figure 1).

**Table 1:** Clinical data analysis.

	S-MCI	Stroke control group (S-C)	*p* value
Age, year^a^	53.33 ± 5.98	53.30 ± 7.39	>0.05
Edu, year^a^	10.37 ± 5.29	10.37 ± 5.94	>0.05
Gender, *n* (M/F)	31 (15/16)	31 (17/14)	>0.05
MMSE^b^	20.00 ± 2.03	28.00 ± 1.64	<0.001
MoCA^b^	20.37 ± 1.79	28.37 ± 1.67	<0.001

Data are mean ± standard deviation unless otherwise stated. ^a^Two-sample *t-*test. ^b^Mann–Whitney *U* test. MMSE, Simple Intelligence scale; MoCA, Montreal Cognitive Scale.

### 3.2 ALFF changes in S-MCI patients

**Figure 2:**
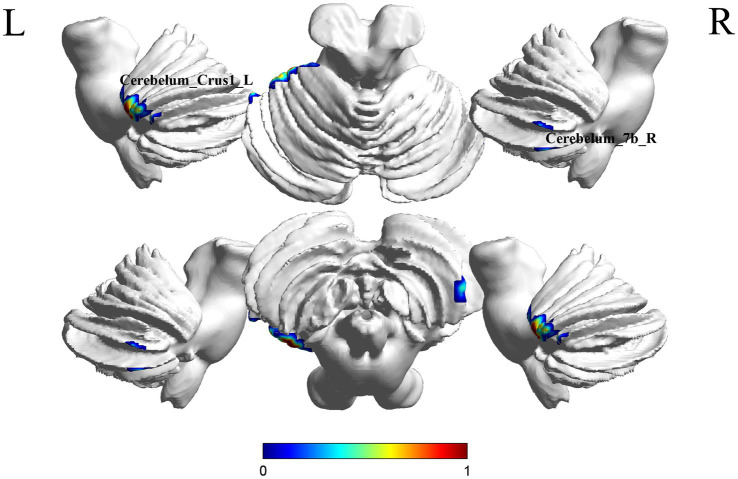
Brain regions (Cerebelum_7b_R, Cerebelum_Crus1_L) showing significant ALFF in the S-MCI group compared with the S-C group on the 3D template (*p* < 0.05, cluster Level FWE correction). The color scale represents the *t*-value. R = right, L = left.

A comparison between the Stroke-MCI group and the stroke-C group showed significant differences in 2 brain regions between the two groups (*p* < 0.05, cluster Level FWE correction, Table 2). ALFF value was increased in Cerebelum_7b_R, Cerebelum_Crus1_L (Figure 2 and Supplementary Figure S1).

**Table 2:** Brain region differences between S-MCI and S-C based on ALFF.

Brain regions^a^	Voxel (mm^3^)	AAL	MNI coordinates			*T-*value
			x	y	z	
S-MCI > S-C						
Cluster 1	40					
Cerebelum_7b_R	10	102	45	−57	−54	6.34
Cluster 2	135					
Cerebelum_Crus1_L	18	91	−48	−39	−33	6.13

^aThe brain area selects the peak point of the mass.^

### 3.3 fALFF changes in S-MCI patients

**Figure 3:**
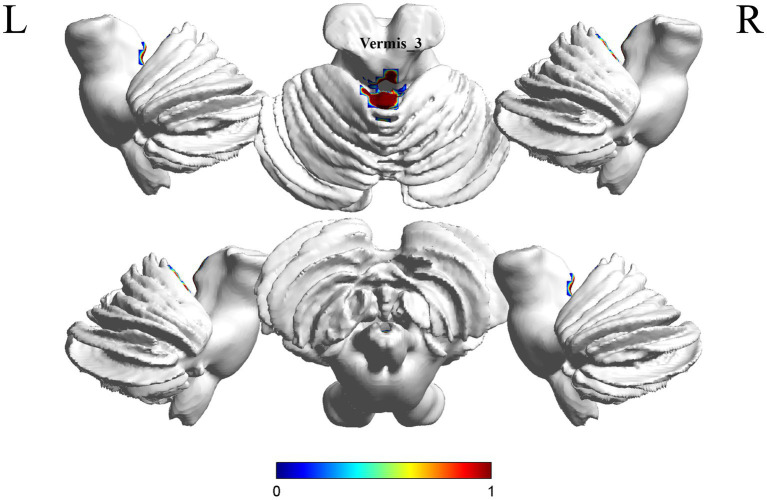
Brain regions (Vermis_3) showing significant fALFF in the S-MCI group compared with the S-C group on the 3D template (*p* < 0.05, cluster Level FWE correction). The color scale represents the *t*-value. R = right, L = left.

A comparison of the S-MCI group with the S-C group showed a difference in 1 brain region between the two groups (*p* < 0.05, cluster Level FWE correction, Table 3). fALFF values was increased in Vermis_3 (Figure 3 and Supplementary Figure S2).

**Table 3:** Brain region differences between S-MCI and S-C based on fALFF.

Brain regions^a^	Voxel (mm^3^)	AAL	MNI coordinates			*T*-value
			x	y	z	
S-MCI > S-C						
Cluster 1	76					
Vermis_3	30	110	0	−42	−3	4.87

^aThe brain area selects the peak point of the mass.^

### 3.4 ReHo changes in S-MCI patients

**Figure 4:**
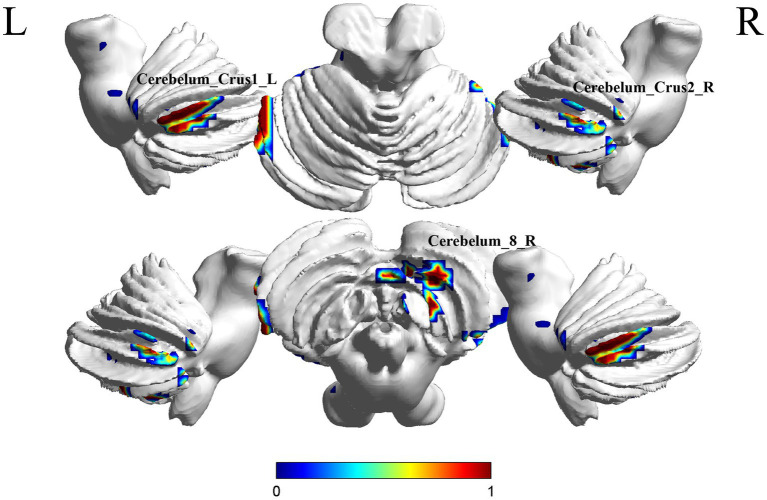
Brain regions (Cerebelum_8_R, Cerebelum_Crus2_R, Cerebelum_Crus1_L) showing significant ReHo in the S-MCI group compared with the S-C group on the 3D template (*p* < 0.05, cluster Level FWE correction). The color scale represents the *t*-value. R = right, L = left.

A comparison between the S-MCI group and the S-C group showed significant differences in 3 brain regions between the two groups (*p* < 0.05, cluster Level FWE correction, Table 4). The ReHo value was increased in Cerebelum_8_R, Cerebelum_Crus2_R, Cerebelum_Crus1_L (Figure 4 and Supplementary Figure S3).

**Table 4:** Brain region differences between S-MCI and S-C based on ReHo.

Brain regions^a^	Voxel (mm^3^)	AAL	MNI coordinates			*T* value
			x	y	z	
S-MCI > S-C						
Cluster 1	100					
Cerebelum_8_R	89	104	21	66	54	4.56
Cluster 2	272					
Cerebelum_Crus2_R	53	94	45	−36	−39	6.89
Cluster 3	651					
Cerebelum_Crus1_L	138	91	−51	−45	−36	6.31

^aThe brain area selects the peak point of the mass.^

### 3.5 Changes of ROI-wise FC in S-MCI patients

**Figure 5:**
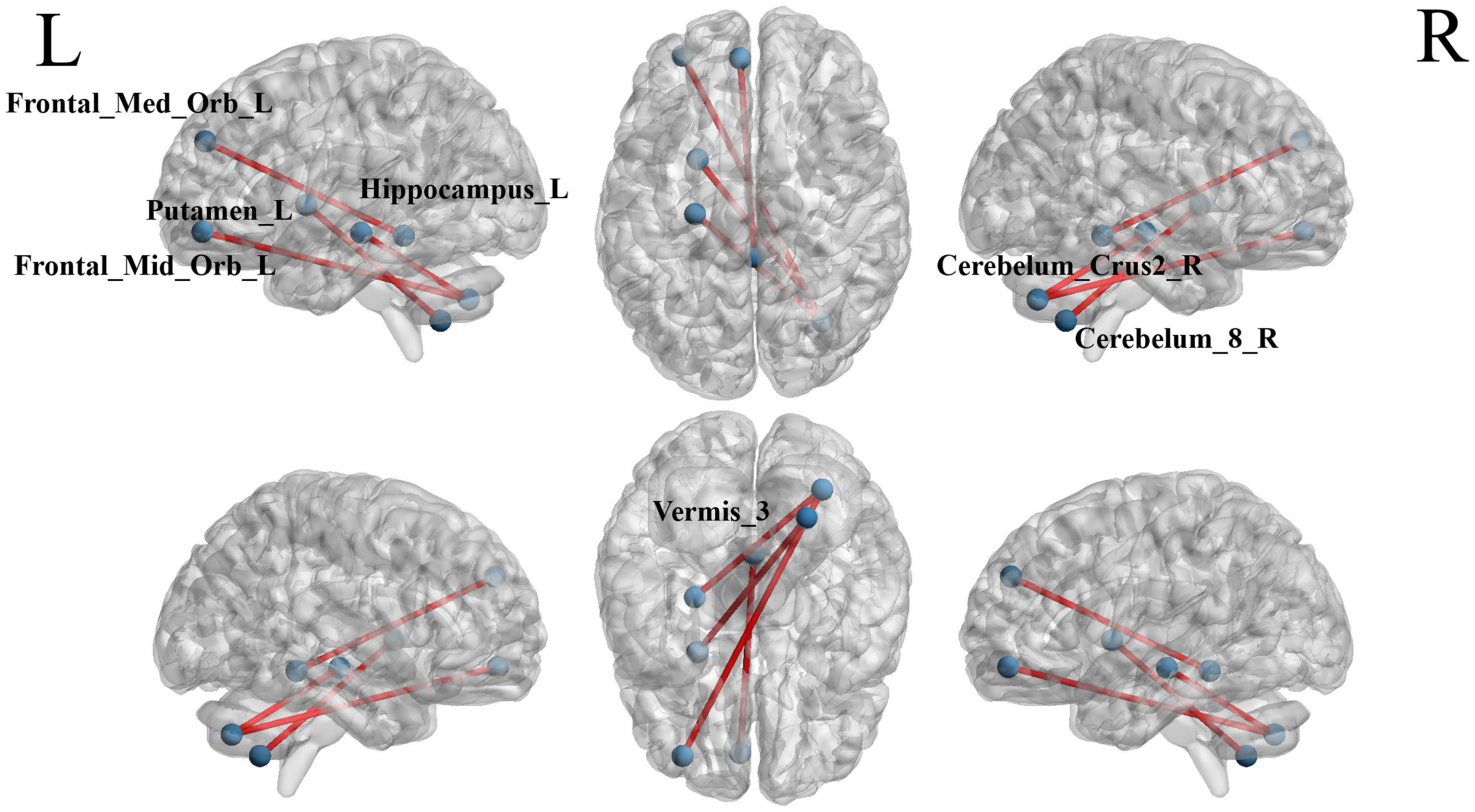
The blue dots represent brain ROI, and the red lines represent brain regions that show a decline in functional brain connectivity (*p* < 0.000999001, NBS-corrected). R = right, L = left.

The ROI-wise FC comparison showed significant differences in three brain regions between the two groups (*p* < 0.000999001, NBS correction, Table 5). The functional connection between Frontal_Mid_Orb_L and Cerebelum_Crus2_R brain regions was decreased, the functional connection between Hippocampus_L and Cerebelum_Crus2_R brain regions was decreased. Functional connection between Vermis_3 and Frontal_Med_Orb_L brain regions was decreased (Figure 5).

**Table 5:** Difference of edge analysis based on ROI-wise FC (seed-seed).

ROI (seed)	ROI (seed)	*P*-value
Cerebelum_Crus2_R	Frontal_Mid_Orb_L	0.0004
	Hippocampus_L	0.0009
Vermis_3	Frontal_Med_Orb_L	0.0006
Cerebelum_8_R	Putamen_L	0.0002

## 4 Discussion

In this study, we conducted analyses of ALFF, fALFF, ReHo, and ROI-wise FC in the cerebellar regions of patients with PSCI and examined the correlation between these neuroimaging changes and cognitive abilities. The findings revealed that stroke patients with cognitive impairment exhibited alterations in ALFF, fALFF, ReHo, and ROI-wise FC across multiple brain regions, including Cerebelum_7b_R, Cerebelum_Crus1_L, and Vermis_3. The observed increases in ALFF and fALFF indicated abnormal neuronal activity levels within these specific brain areas, suggesting a state of overactivation among neurons. Conversely, reductions in these parameters reflected diminished overall neuronal activity within the corresponding regions. Meanwhile, variations in ReHo values provided insights into changes in local neuronal synchronization. Furthermore, at the level of functional connectivity within the cerebellar-limbic system, we identified alterations in the strength of connections between various brain functions among patients suffering from PSCI. These connections may be either enhanced or weakened-further indicating disruptions to the coordination mechanisms between different brain regions under this pathological condition. This impaired collaborative ability is likely to be a significant factor contributing to cognitive dysfunction observed in stroke patients.

Individuals with cognitive impairment may exhibit alterations in the function or structure of the cerebellum (Jacobs et al., 2018). We assessed neuronal activity levels within the cerebellar region using ALFF, fALFF, and ReHo methodologies, discovering that stroke patients with cognitive impairment displayed significant changes in the activity of certain brain regions. Furthermore, research conducted by Rudolph et al. (2023). Highlighted the critical role of the vermis in emotion regulation, particularly regarding how its stimulation influences emotional responses. Concurrently, Yin et al. (2021) emphasized that alterations involving cerebellar vermis I and II were closely associated with patients’ cognitive abilities. This relationship might be reflected through FC. In our study, we specifically concentrated on changes in the activity of cerebellar vermis III, which were quantitatively analyzed utilizing ALFF, fALFF, and ReHo techniques. At the same time, we applied identical analytical methods to other areas within the cerebellum’s maxillary region. The results indicated an increase in ReHo for Cerebelum_8_R, Cerebelum_Crus2_R, Cerebelum_Crus1_L among other regions. Notably, there was also an increase observed in ALFF for Cerebelum_7b_R and Cerebelum_Crus1_L as well as a rise in fALFF for Vermis_3. Moreover, these changes exhibited a strong correlation with cognitive impairment. This finding underscored the significant role of the cerebellum in cognitive functioning.

Regarding the mechanisms through which the cerebellum regulates emotional signals, it has been established that the cerebellum plays a significant role in modulating emotional signals and behaviors via its extensive cerebellar-cortical and subcortical circuits (Verpeut et al., 2023). The cerebellar vermis and nuclei may contribute to memory processing by transmitting or prediction error signals through connections with various cortical and subcortical structures, including the prefrontal cortex, amygdala, parabrachial nucleus, and PAG (Van Overwalle et al., 2020; Wagner et al., 2021). Song et al. (2025) demonstrated a significant reduction in FC within the prefrontal cortex and the posterior cerebellar lobe among adolescents with schizophrenia. This reduction was positively correlated with performance on simple visuospatial memory tests, suggesting that alterations in the prefrontal cortex-posterior cerebellar circuit may impact cognitive function (Song et al., 2025). Similarly, diminished connectivity in the prefrontal cortex-cerebellar circuit has been observed in individuals with autism, leading to a decline in executive functions, including social information processing (Attanasio et al., 2024). Hua et al. (2024) further proposed that the prefrontal cortex and cerebellum are likely interconnected via neural networks, contributing jointly to various functions such as pain perception, emotional regulation, cognitive processing, and motor control. This underscores the intricate involvement of the cerebellum in cognitive functions.

The cerebellum is a crucial component of the brain network involved in social cognition. Both the cerebellum and other regions of the brain play significant roles in cognitive processing. However, the mechanisms by which they collaborate to address cognitive impairment remain to be elucidated. Simultaneously, there exists ongoing debate regarding brain-cerebellar connectivity. Qi et al. (2019) argued that this connectivity diminished, while Tang et al. (2021) contended that it increased. In this study, we employed rs-fMRI to examine differences in functional connectivity between the limbic system and the cerebellum among patients with and without PSCI, as well as identifying brain regions within the cerebellum that exhibit functional changes during episodes of cognitive impairment. This research aims to enhance our understanding of the critical role played by the cerebellum in cognitive dysfunction. Previous studies have demonstrated functional connections between the cerebellum and various cognition-related areas within the cerebral cortex among stroke patients experiencing cognitive impairments. These connections imply that the cerebellum may participate in regulating cognitive processing through intricate neural circuits, thereby providing evidence for its involvement in such functions (Ruan et al., 2023). Our findings align with these observations. Specifically, we noted a reduction in functional connectivity between the limbic system and structures such as the vermis. Building on this discourse, Li et al. (2023) proposed a model of functionally corresponding partitioned connections and discovered that connectivity decreased among functionally corresponding regions while increasing among non-corresponding ones. Additionally, some studies have identified the cerebellum as a key region implicated in language processing and overall cognitive function (Stilling et al., 2024). Meanwhile, our study confirmed that the connectivity between the cerebellum and limbic brain regions associated with cognitive memory, such as the hippocampus, was diminished. In accordance with the majority of prior studies and corroborated by the findings of this investigation, patients with MCI exhibit a marked diminution in functional connectivity between the cerebellum and cerebral regions. This alteration is likely to contribute to the observed decline in cognitive functions. We hypothesize that this change in functional connectivity may represent a primary factor underlying the dysfunction experienced by MCI patients. Furthermore, the diminished coordination between the brain and cerebellum in MCI patients may constitute a critical pathogenic mechanism contributing to cognitive impairment.

The limbic system comprises several key structures, including the parahippocampal gyrus, cingulate gyrus, subcallosal region, hippocampus and dentate nuclei, superior callosal gyrus (also referred to as the dorsal aspect of the corpus callosum), paratropular gyrus, olfactory tract and bulb, hypothalamus, among others (Tien et al., 1994). Each component serves a distinct function and is intricately interconnected with other parts of the system. Both the limbic system and subcortical nuclei are integral to emotional expression and play a significant role in the pathophysiology of neuropathological conditions such as affective disorders (Naidich et al., 1987). Research has demonstrated that both the hippocampus and limbic system are closely associated with memory formation and consolidation while also being crucial for emotional development and expression. Rondi-Reig et al. (2022) proposed that there exists a bidirectional communication mechanism between the cerebellum and hippocampus. In studies examining whole-brain connectivity with the hippocampus as the seed region, patients with MCI exhibited significantly reduced connectivity in the cerebellar Crus II (Jiang et al., 2022). Similarly, in patients with AD-MCI, the investigators observed significantly reduced DFC in the white matter of both the left and right hippocampi, as well as in the left cerebellar crus 2 (Cerebelum_9_L) (Wang et al., 2022). We observed a reduction in the connectivity between the cerebellum and the hippocampus in patients with MCI. This finding suggests that the cerebellar-hippocampal connections play a critical role in higher-order cognitive functions. We hypothesize that, given the hippocampus’s pivotal role in memory processing, diminished connectivity between these two brain regions primarily impairs memory function in MCI patients, thereby contributing to overall cognitive decline. Specifically, this interaction allows the cerebellum to modulate cognitive processes such as learning and memory by influencing hippocampal activity.

Studies have indicated that stimulating the anterior cerebellum can effectively activate multiple brain regions, including the orbitofrontal cortex, hippocampus, and posterior hypothalamus (Badura et al., 2018). In our investigation of stroke patients with cognitive impairment, we observed a notable enhancement in neuronal activity within the hippocampus, aligning with the findings reported by Zhang et al. (2021). The hippocampus is primarily responsible for various cognitive functions such as memory processing and storage, as well as spatial information processing (Kumaran, 2005). The relationship between the hippocampus and human memory has been extensively validated in earlier research. Moreover, Tao’s task-related functional magnetic resonance imaging studies involving patients with MCI also identified abnormal activity in the hippocampus during the memory encoding phase (Tao et al., 2019). Collectively, these findings lead to an important conclusion that heightened arousal states within the hippocampus may be a key factor contributing to cognitive deficits. We hypothesize that stimulation of the forecerebellum induces hyperactivity in the hippocampus, which may disrupt normal memory processing and information storage mechanisms-ultimately resulting in cognitive impairment. This supports their collaborative role in addressing cognitive dysfunctions. The role of the cerebellum in PSCI has historically been underappreciated. The findings of this study may offer a novel avenue for advancing PSCI rehabilitation. By monitoring the atypical activity of the cerebellar limbic system post-stroke, a personalized intervention framework can be established to modulate cerebellar activity, thereby enhancing the efficacy of cognitive rehabilitation practices following stroke.

However, this study has certain limitations as it is a pilot study. Firstly, the limited sample size coupled with the presence of confounding factors, such as medication usage, may compromise the accuracy and reliability of the results. Secondly, we did not correlate the changes in specific brain regions with alterations across multiple functional networks. Finally, our study primarily concentrates on the alterations in cerebellar activity, with limited exploration of the cerebellar network. Future research endeavors will encompass a broader range of cases and employ advanced analytical techniques to investigate both intrinsic cerebellar abnormalities and the connectivity within the cerebellar-brain system.

## 5 Conclusion

In conclusion, our study identified abnormalities in the cerebellar region and the cerebellar-brain system among patients with post-stroke cognitive impairment. There was a disruption in functional connectivity between the cerebellum and limbic system of the patients, with the extent of this impairment correlating with levels of cognitive dysfunction. Additionally, abnormalities were observed in neuronal activity within these brain regions. These altered indicators might contribute to diminished cognitive function and impaired decision-making abilities, potentially representing a significant mechanism underlying cognitive impairment following early stroke.

## Data Availability

The original contributions presented in the study are included in the article/Supplementary material, further inquiries can be directed to the corresponding authors.

## Footnotes

^1^ https://rest-fmri.net/forum/RESTplus

^2^ http://www.fil.ion.ucl.ac.uk/spm/software/spm12/

^3^ https://www.nitrc.org/projects/gretna
