# Corrigendum: Abnormal cerebellar activity and connectivity alterations of the cerebellar-limbic system in post-stroke cognitive impairment: a study based on resting state functional magnetic resonance imaging

**DOI:** 10.3389/fnins.2025.1620381

**Published:** 2025-05-13

**Authors:** Haiyi Zhang, Juan Lu, Lu Zhang, Jidan Hu, Jiajun Yue, Yunhan Ma, Qi Yao, Pingping Jie, Min Fan, Jiliang Fang, Jie Zhao

**Affiliations:** ^1^Department of Magnetic Resonance Imaging, The Affiliated Traditional Chinese Medicine Hospital, Southwest Medical University, Luzhou, Sichuan, China; ^2^Department of Acupuncture and Rehabilitation, The Affiliated Traditional Chinese Medicine Hospital, Southwest Medical University, Luzhou, Sichuan, China; ^3^Department of Radiology, The Second People's Hospital of Neijiang, Southwest Medical University, Neijiang, Sichuan, China; ^4^Department of Nuclear Medicine, The Affiliated Hospital of Southwest Medical University, Luzhou, China; ^5^Molecular Imaging Key Laboratory of Sichuan Province, Department of Nuclear Medicine, The Affiliated Hospital of Southwest Medical University, Luzhou, China; ^6^Department of Radiology, The Affiliated Traditional Chinese Medicine Hospital, Southwest Medical University, Luzhou, China; ^7^Guang'anmen Hospital, China Academy of Chinese Medical Sciences, Beijing, China

**Keywords:** stroke, mild cognitive impairment, cerebellum, functional magnetic resonance imaging, limbic system

In the published article, there was an error in affiliation 1. Instead of “^1^Department of Magnetic Resonance Imaging, The Affiliated Traditional Chinese Medicine Hospital, Luzhou, Sichuan, China”, it should be “^1^Department of Magnetic Resonance Imaging, The Affiliated Traditional Chinese Medicine Hospital, Southwest Medical University, Luzhou, Sichuan, China”.

The authors apologize for this error and state that this does not change the scientific conclusions of the article in any way. The original article has been updated.

