# Supplementary material for: Abnormal cerebellar activity and connectivity alterations of the cerebellar-limbic system in post-stroke cognitive impairment: a study based on resting state functional magnetic resonance imaging
Source: Front Neurosci. 2025 Mar 19;19:1543760. doi: 10.3389/fnins.2025.1543760 (PMC11962788; doi:10.3389/fnins.2025.1543760)
Supplement: Supplementary file 1 [file Data_Sheet_1.pdf]

frontiers

Supplementary Material

Abnormal cerebellar activity and connectivity alterations of the cerebellar-limbic system in post-stroke cognitive impairment: A study based on resting state functional magnetic resonance imaging Contents

Figure S1. Brain regions (Cerebelum_7b_R, Cerebelum_Crus1_L) showing significant ALFF in the S-MCI group compared with the S-C group on the 3D template (P < 0.05, GRF corrected). The color scale represents the t-value. R = right, L = left.

Figure S2. Brain regions (Vermis_3) showing significant fALFF in the S-MCI group compared with the S-C group on the 3D template (P < 0.05, GRF corrected). The color scale represents the t-value.R = right, L = left.

Figure S3. Brain regions (Cerebelum_8_R, Cerebelum_Crus2_R, Cerebelum_Crus1_L) showing significant Reho in the S-MCI group compared with the S-C group on the 3D template (P < 0.05, GRF corrected). The color scale represents the t-value. R = right, L = left.

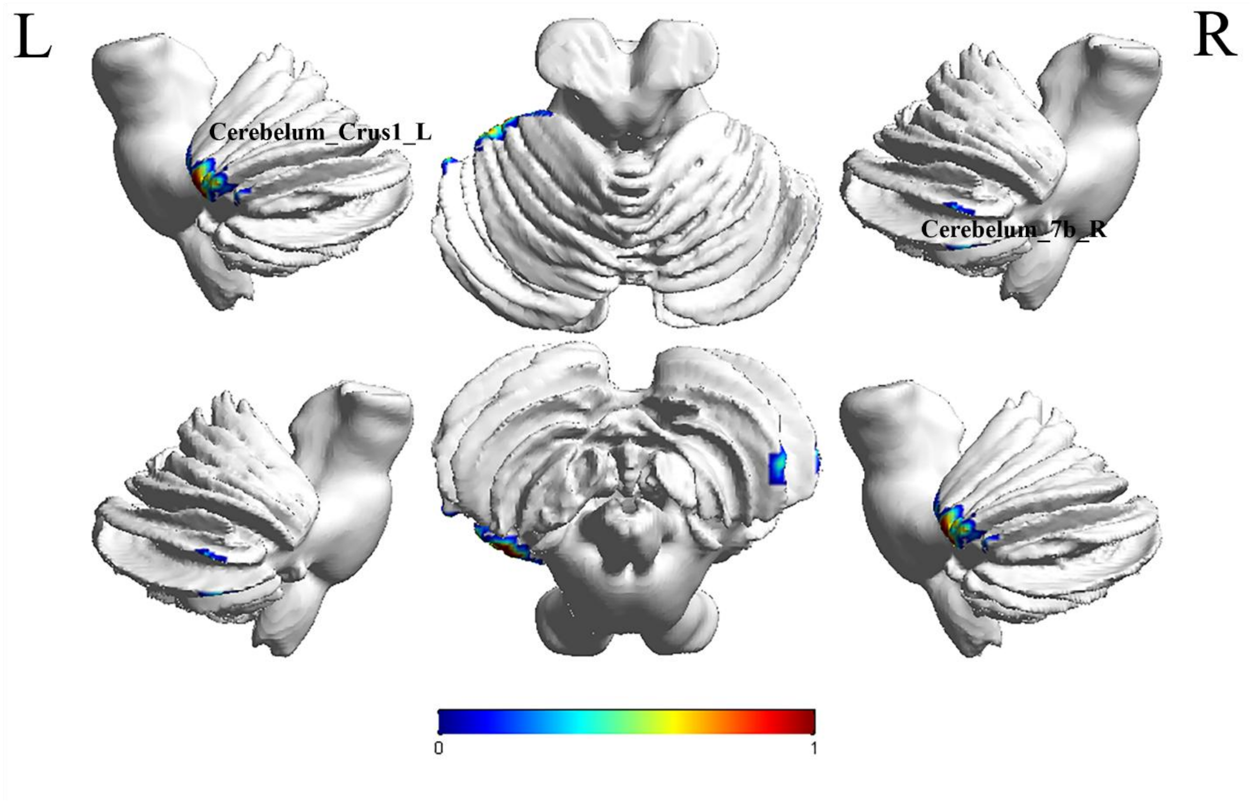

Figure S1. Brain regions (Cerebelum_7b_R, Cerebelum_Crus1_L) showing significant ALFF in the S-MCI group compared with the S-C group on the 3D template (P < 0.05, GRF corrected). The color scale represents the t-value. R = right, L = left.

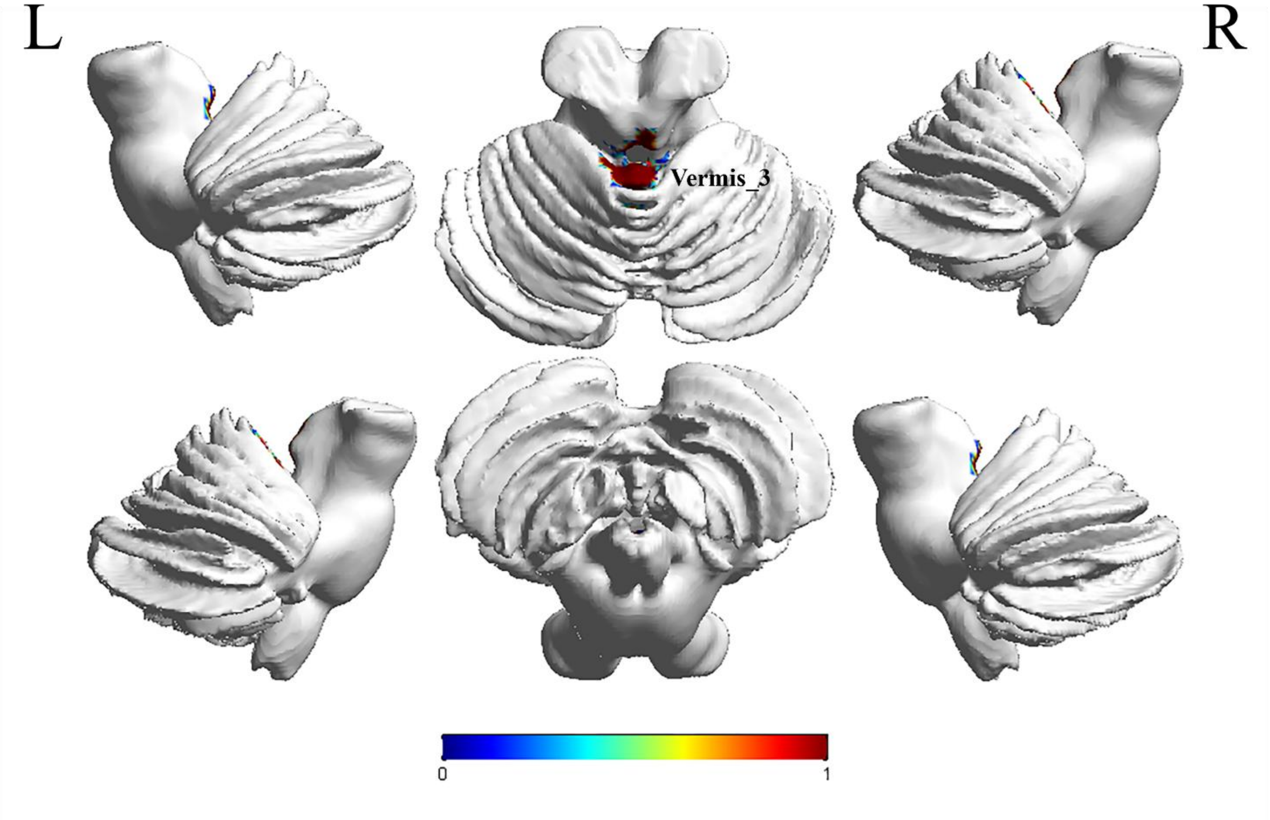

Figure S2. Brain regions (Vermis_3) showing significant fALFF in the S-MCI group compared with the S-C group on the 3D template ($P < 0.05$, GRF corrected). The color scale represents the t-value.R = right, L = left.

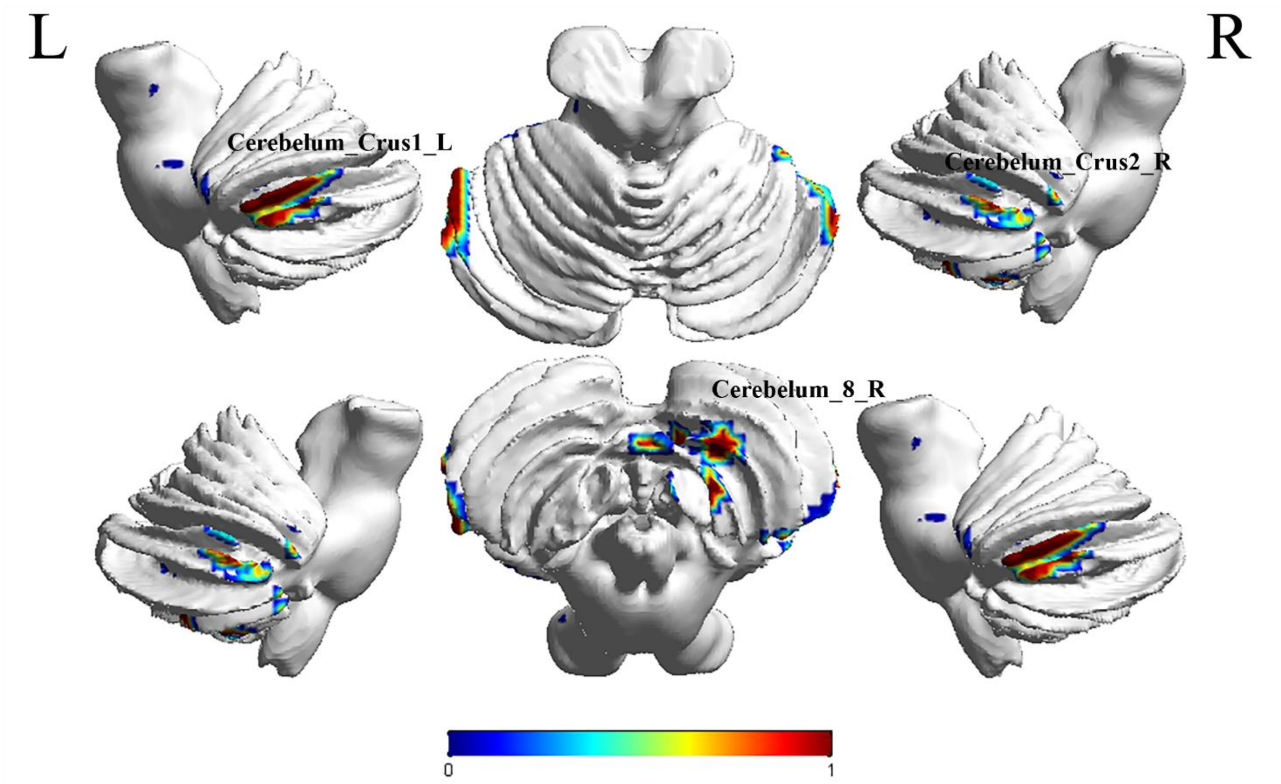

Figure S3. Brain regions (Cerebelum_8_R, Cerebelum_Crus2_R, Cerebelum_Crus1_L) showing significant ReHo in the S-MCI group compared with the S-C group on the 3D template (P < 0.05, GRF corrected). The color scale represents the t-value. R = right, L = left.
